# The biobehavioural pain and movement questionnaire (BioPMovQ): development and psychometric validation of a new questionnaire

**DOI:** 10.3389/fmed.2024.1358829

**Published:** 2024-05-09

**Authors:** Roy La Touche, Alba Paris-Alemany, Joaquín Pardo-Montero, Diego Miñambres-Martín, Francisco Mercado-Romero, Irene de la Rosa-Díaz, Miguel A. Sorrel, Mónica Grande-Alonso

**Affiliations:** ^1^Departamento de Fisioterapia, Centro Superior de Estudios Universitarios (CSEU) La Salle, Universidad Autónoma de Madrid, Madrid, Spain; ^2^Motion in Brains Research Group, Institute of Neuroscience and Sciences of the Movement (INCIMOV), Centro Superior de Estudios Universitarios La Salle, Madrid, Spain; ^3^Instituto de Dolor Craneofacial y Neuromusculoesquelético (INDCRAN), Madrid, Spain; ^4^PhD Program in Medicine and Surgery, Doctoral School, Universidad Autónoma de Madrid, Madrid, Spain; ^5^Departamento de Radiología, Rehabilitación y Fisioterapia, Facultad de Enfermería, Fisioterapia y Podología, Universidad Complutense de Madrid, Madrid, Spain; ^6^La Paz Hospital Institute for Health Research, IdiPAZ, Madrid, Spain; ^7^Premium Madrid Global Health Care, Madrid, Spain; ^8^Faculty of Sport Sciences, Universidad Europea de Madrid, Madrid, Spain; ^9^Cognitive Neuroscience, Pain, and Rehabilitation Research Group (NECODOR), Faculty of Health Sciences, Rey Juan Carlos University, Madrid, Spain; ^10^Department of Psychology, Faculty of Health Sciences, Rey Juan Carlos University, Madrid, Spain; ^11^Departamento de Psicología Social y Metodología, Universidad Autónoma de Madrid, Madrid, Spain; ^12^Departamento de Cirugía, Ciencias Médicas y Sociales, Facultad de Medicina, Universidad de Alcalá, Alcalá de Henares, Spain; ^13^Grupo de Investigación Clínico-Docente sobre Ciencias de la Rehabilitación (INDOCLIN), Centro Superior de Estudios Universitarios La Salle, Madrid, Spain

**Keywords:** chronic pain, biobehavioural approach, self-administered questionnaires, psychological variables, functionality

## Abstract

**Objective:**

The purpose of this research was to design and psychometrically validate a new instrument (the Biobehavioural Pain and Movement Questionnaire/BioPMovQ), which assesses the relationship between pain and various factors related to motor behaviour from a biobehavioural perspective.

**Methods:**

A mixed-method design combining a qualitative study with an observational and cross-sectional study was employed to develop (content validity) and psychometrically validate (construct validity, reliability and concurrent/discriminant validity) a new instrument. A total of 200 patients with chronic musculoskeletal pain were recruited.

**Results:**

According to the exploratory factor analysis, the final version of the BioPMovQ consists of 16 items distributed across 4 subscales (1, disability, 2, self-efficacy for physical activity; 3, movement avoidance behaviours; and 4, self-perceived functional ability), all with an eigen value greater than 1, explaining 55.79% of the variance. The BioPMovQ showed high internal consistency (Cronbach’s *α* = 0.82; McDonald’s *ω* = 0.83). The intraclass correlation coefficient was 0.86 (95% confidence interval 0.76 to 0.91), which was considered to demonstrate excellent test–retest reliability. The standard error of measurement and minimal detectable change were 3.43 and 8.04 points, respectively. No floor or ceiling effects were identified. There was a positive, significant and moderate magnitude correlation with the Graded Chronic Pain Scale (*r* = 0.54), kinesiophobia (*r* = 0.60), pain catastrophising (*r* = 0.44) and chronic pain self-efficacy (*r* = −0.31).

**Conclusion:**

The BioPMovQ showed good psychometric properties. Based on the findings of this study, the BioPMovQ can be used in research and clinical practice to assess patients with chronic musculoskeletal pain.

## 1 Introduction

Pain is a complex multidimensional experience that significantly influences behaviour and has a perceptual character dependent on context and individual evaluative processes. In the multidimensional aspect of pain, movement is considered to play a key role (1). Simmonds et al. (2) considered that movement responses are not just a consequence of anticipating and minimising pain; the authors’ proposed that the motor behaviour involved in pain is a very complex factor because psychological (cognition and emotions), social and contextual factors can influence motor activity within the behavioural component of pain as a multidimensional experience (2). Many psychological constructs are significant predictors of outcomes, such as pain, disability and work retention (3). These variables are relevant in a biobehavioural approach.

Several studies have reported the onset of functional and structural changes in the cortical motor areas of patients with chronic pain (4, 5). Pain-related movement disorders have been proposed to neurophysiologically involve central and peripheral mechanisms, which have varying degrees of influence in determining behavioural performance (6). There are also numerous studies on kinematic abnormalities associated with musculoskeletal pain (7–9).

In the past 2 decades, several theories have been developed to explain the relationship between pain and movement (10), which have been designed on the basis of findings from studies of experimentally induced pain (11) and on research assessing functional deficits and disability through the influence or interaction of sensory, cognitive, emotional and motor components in patients with chronic pain (12–16).

A number of authors have suggested the general premise that pain generates changes in the way patients move, which in turn can change the way patients perceive the painful experience (17–19), leading to the proposition that movement is initially involved in the experience of pain as an adaptive and protective response to control or diminish its perception (10, 20). These evoked responses can influence movement by altering neuromuscular speed, variability and efficiency. The theory of the motor behaviour dimension of the pain experience encompasses the set of adaptive or maladaptive motor responses related to the pain experience that affect modulation, processing and function and that also interact with or are influenced by contextual, cognitive and affective-motivational factors (1).

Emotional factors related to fear of pain play an important role in the degree of protective behaviours experienced when faced with pain (21). Research has shown that extreme fear of pain is associated with being less physically active (22, 23), having limited range of motion (24, 25), having greater physical disability (26) and developing strategies for adopting alternative movements (27). Behaviours associated with psychological distress, activity disruption and activity avoidance are essential components of pain-related disability (28).

Current evidence supports the fact that psychosocial factors other than fear of pain might contribute to pain-related functional impairment (29–31). Sullivan et al. suggested that certain psychological factors, such as pain catastrophising, fear and depression can influence pain behaviour by lowering the threshold for activating motor programmes related to the experience of pain (21).

Although more research is still needed to determine the complex interactions between movement and pain and their mutual influence on cognitive, behavioural and social factors, it is important to understand the relationship between movement and pain (32). It is now considered a clinical necessity to evaluate these interactions from a biobehavioural perspective, taking into account that pain-related movement disorders have a significant effect on the deterioration of the patient’s functional capacity and quality of life (33).

Biobehavioural factors related to pain, functional limitation and disability can be classified into three broad categories: (1) cognitive-perceptual (cognitive-perceptual bias, perceived control, perceived disability, fear of pain, work and family perceptions and perceived self-efficacy); (2) behavioural-environmental (positive and negative behavioural consequences and physical stressors); and (3) physiological (physiological responses to work and physiological responses to pain or other aversive somatic stimuli) (33).

The central hypothesis of this research posits that the Biobehavioural Pain and Movement Questionnaire (BioPMovQ), specifically developed to evaluate the multifaceted impact of pain on movement from a biobehavioural standpoint, will demonstrate robust validity and psychometric properties, rendering it suitable for application in individuals suffering from musculoskeletal pain.

In light of the intricate interplay between pain, motor behaviour, and psychosocial factors in chronic musculoskeletal pain management, this study aims to address three primary objectives, each responding to a distinct need highlighted in the literature.

The first objective is to design a self-administered instrument assessing the impact of pain on various factors related to motor behaviour from a biobehavioural perspective. There are currently no valid and reliable assessment instruments that assess the multidimensional influence of pain on movement from a biobehavioural perspective. A single instrument that assesses various factors related to pain and movement (such as exercise self-efficacy, avoidance behaviours, physical discomfort, disability and perceived functional ability) could be useful for clinicians involved in the study and treatment of pain.

The second objective, to evaluate the comprehension and content validity of the designed instrument, is rooted in the necessity to ensure that the instrument is both intelligible to patients and clinically relevant. This is pivotal to ensure that the measurements accurately reflect patients’ experiences and are useful for health practitioners in clinical decision-making.

Lastly, the third objective aims to identify the basic psychometric properties of the instrument. This objective addresses the critical need for reliable and valid assessment tools to measure the complexities of musculoskeletal pain and its effects on motor behaviour. Psychometric validation is indispensable for advancing both research and clinical practice, enabling more tailored and effective interventions.

## 2 Methods

A mixed method design combining a qualitative study with an observational and cross-sectional study was employed to develop and psychometrically validate the new instrument. The design of the BioPMovQ was developed using a standardised methodology based on six phases (34): (1) perform an intensive literature review; (2) perform semi-structured interviews; (3) synthesise the literature review and analyse the semi-structured interviews; (4) develop the items (detail the items and identify the domains); (5) perform expert validation (content validity); and (6) assess the instrument’s comprehension and feasibility (cognitive debriefing) in a small group of patients (pilot testing). The procedures during the psychometric validation were performed according to the COSMIN Study Design checklist for patient-reported outcome measurement instruments (35).

The study was approved by the bioethics committee of the Centro Superior de Estudios Universitarios La Salle (CSEULS-PI-005/2020). The objective of the research was explained to all participants in detail, who provided written informed consent to participate in the study. The data were collected between January 2020 and February 2022.

### 2.1 Development of the items

#### 2.1.1 Literature review

A search of the scientific literature was performed in 6 specialised databases (Medline, PEDro, PsycINFO, CINAHL, EMBASE and Web of Science). Information was extracted from narrative reviews, qualitative studies, observational studies, systematic reviews and meta-analyses addressing the topics of pathophysiology of musculoskeletal pain, neurophysiology of chronic pain, pain-related motor impairments, function and disability, assessment of pain and movement and the social and psychological implications related to disability and functional ability.

The content analysis of the scientific literature was independently evaluated by 2 researchers who performed a tabular extraction of the relevant topics.

A second search was conducted to identify psychometrically validated self-report instruments aimed at identifying motor and functional implications related to pain, disability and psychosocial aspects. A total of 17 self-reporting instruments were identified and analysed in depth from a critical perspective, taking into account each instrument’s differences, similarities, advantages, disadvantages and limitations (36–52).

#### 2.1.2 Semi-structured interviews

Based on the scientific literature review, a semi-structured interview for patients with chronic musculoskeletal pain was designed by consensus. The interview was developed by focusing on the relationship between pain and movement function and possible psychosocial interference. Drafts of the interview questions were discussed and reviewed by the research team during a pre-established session. A semi-structured interview was conducted involving 12 patients with chronic musculoskeletal pain.

#### 2.1.3 Synthesis of the literature review and semi-structured interview analysis

The results of the semi-structured interview were analysed, employing an interpretative phenomenological analysis (53), which is a qualitative study that assesses the meaning that people attach to their own experiences (54–56).

From the qualitative analysis, it was possible to extract the experiences and a construct meaning, and a list of three main themes and seven sub-themes was generated, which included (1) behaviours and cognitions of fear-avoidance; (2) pain-related changes in motor behaviours; (3) coping behaviours; (4) pain-related disability; (5) pain-related bodily perceptions; (6) self-efficacy related to activities of daily living; and (7) exercise self-efficacy.

The final analysis of this phase was performed by 2 researchers who, through a consensus methodology, grouped and sorted the results of the qualitative study and literature review.

From the synthesis of scientific evidence obtained through literature review, five fundamental conclusions can be drawn:

1. A multitude of studies have delineated or hypothesised various neurophysiological mechanisms, both central and peripheral, associated with pain. These mechanisms might be linked to alterations in movement and functionality, such as changes in motor control, range of movement, muscle strength and endurance, as well as the onset of disability.
2. Various factors influencing motor behaviour associated with pain—such as disability, avoidance behaviours, movement fear, and deficits in self-efficacy—can significantly impact patient quality of life. These factors are interconnected with multiple psychological aspects.
3. Disability is identified as a crucial factor in the analysis of patients with chronic pain, highlighting how these individuals face challenges in performing daily activities, recreational activities, physical activities, and work-related tasks.
4. Motor and functional alterations associated with pain could be conceptualised as a multidimensional construct that encompasses cognitive, emotional, sensory, and social dimensions.
5. Currently, there are no psychometrically validated instruments available that comprehensively assess the implications of motor and functional alterations associated with pain from a multidimensional perspective. There are various instruments that specifically and separately evaluate disability, self-efficacy in different pain-related contexts (physical activity, exercise, pain self-management) and factors associated with fear and activity avoidance.

#### 2.1.4 Developing items

Findings from the relevant literature and data from the semi-structured interviews were qualitatively analysed by 3 researchers to define the conceptual construct of “pain related to movement and function from a biobehavioural perspective.” Subsequently, the articles were written. A total of 28 items were designed through a structured consensus process (57), with 27 items ultimately established for this phase and ordered by their relevance within each dimension (sub-constructs).

#### 2.1.5 Expert-validated content

A 26-item preliminary list was drafted for the scale, the suitability of which (relevance, pertinence, clarity, coherence and degree of coverage of the relevant aspects) was evaluated by an external expert panel (validation by judges). The requirements to be considered an expert judge were (1) having at least 4 publications related to chronic pain, movement and function; (2) at least 10 years of clinical or scientific experience in the area of pain and movement; and (3) knowledge about the psychometric validation of questionnaires.

The expert content validation panel consisted of 11 expert judges with research and clinical backgrounds (1 medical doctor, 7 physiotherapists and 3 psychologists), who were asked to conduct a qualitative evaluation (relevance, comprehensiveness and comprehensibility) of every item, using a 5-level Likert scale (1, strongly agree; 2, agree; 3, neither agree nor disagree; 4, disagree; and 5, strongly disagree).

To consider an item for deletion, the following performance indicators were considered: (1) mean item score of <0.70 Aiken’s V statistic (58); (2) the behavioural content did not have a generally accepted meaning or definition; (3) the item was ambiguously defined; (4) the content item was irrelevant or repetitive to the purposes of measurement; and (5) whether the qualified judges had agreed that the item had been adequately sampled based on consensus.

#### 2.1.6 Cognitive debriefing

A cognitive debriefing methodology was applied as a qualitative evaluation of the preliminary version of the instrument by a small group of patients (32 patients). The cognitive debriefing was based on the instrument’s evaluation, considering 5 aspects that analyse the completeness, relevance and clarity of expression (59): (1) comprehension of each question; (2) relevance of the information; (3) decision processes (response time, response/abandonment rate); (4) response processes; and (5) general comments.

### 2.2 Psychometric validation

#### 2.2.1 Participants

A consecutive non-probability sample of participants were recruited from 2 physiotherapy clinics. All participants were assessed by physiotherapists with experience and academic training in managing musculoskeletal disorders, and the patients were classified as having chronic musculoskeletal pain. Chronic musculoskeletal pain is defined as “persistent or recurrent pain arising as part of a pathological process that directly affects the bones, joints, muscles or related soft tissues” (60).

Patients were selected if they met all the following criteria: (1) presence of pain of more than 6 months’ duration; (2) a pain intensity greater than or equal to 3 points on the numerical pain rating scale (NPRS); (3) an age of 18 years or older; (4) chronic primary musculoskeletal pain, classified as primary chronic pain (cannot be directly attributed to a known disease or damage process) or as secondary if caused by a disease or process that directly affects the bones, joints, muscles and/or related soft tissues (61); (5) a good understanding of the Spanish language; and (6) not having started physiotherapy or having undergone fewer than 2 treatment sessions.

The exclusion criteria were (1) cognitive impairment; (2) psychiatric limitations that impede participation in the study assessments; (3) inability to grant written informed consent; (4) a history of musculoskeletal trauma (e.g., fracture); (5) postoperative musculoskeletal pain during the previous 6 months; and (6) musculoskeletal pain suspected to originate from neurological (e.g., stroke), neoplastic (e.g., breast cancer) and/or referred pain (e.g., visceral referred pain).

##### 2.2.2 Sample size

The sample size for the psychometric evaluation was specifically established through a theoretical profile based on exploratory factorial analysis. We estimated that the sample size should exceed 200 cases based on a moderate condition where communalities of between 0.40 and 0.70 and at least 2 factors with more than 4 items each are expected (62). This estimate is in line with the methodological criteria of experts who consider that even under ideal conditions, such as obtaining high communalities and well-determined factors, the sample for studies that perform a factorial analysis should exceed 200 cases (62, 63).

For the sample size calculation for the test–retest reliability study, we employed the method described by Walter et al. (64), which is based on estimating the sample size from assumptions of the intraclass correlation coefficient (ICC) result. The minimum acceptable ICC estimated for the test–retest assessments (2 assessments) was P0 = 0.75; however, we expected an ICC higher than P1 = 0.90. Considering a power of 95% (*β* = 0.5) and an alpha error level of 0.05, the study sample size should comprise 45 participants; after estimating possible losses of 15% for the sample, the total recommended sample size is 53 participants. The sample size was calculated with a web calculator (65).

##### 2.2.3 Procedure

After consenting to participate in the study, the recruited participants received a series of self-reports to assess disability-related and other psychological variables, as well as to record demographic characteristics. The self-reports included the preliminary version of the BioPMovQ, the Spanish version of the Chronic Pain Self-Efficacy Scale (CPSS), the Spanish version of the Pain Catastrophising Scale (PCS), the Spanish version of the Tampa Scale for Kinesiophobia (TSK-11) and the Spanish version of the Graded Chronic Pain Scale (GCPS).

The sociodemographic questionnaire collected information on gender, date of birth, marital status, educational level and employment status.

Biobehavioural Pain and Movement Questionnaire (BioPMovQ) (Draft version).

The preliminary version of the BioPMovQ consisted of 25 items and 5 theoretical subscales (factors) that evaluated (1) movement avoidance behaviours; (2) self-efficacy for physical activity; (3) physical discomfort; (4) self-perceived functional ability; and (5) disability. The items were scored on a 5-level Likert scale (1, strongly agree; 2, agree; 3, neither agree nor disagree; 4, disagree; and 5, strongly disagree). Higher scores indicate greater implications for pain-related motor and functional impairment.

### 2.3 Data analysis

The theoretical construct was determined and its reliability and external validity evaluated using SPSS software version 21 (IBM SPSS Statistics). Descriptive statistics were employed to summarise the data for categorical variables as absolute (number) and relative frequencies (percentage). Sociodemographic and clinical variables are presented as mean ± standard deviation (SD), 95% confidence interval, range (minimum-maximum), Skewness and Kurtosis. A normality analysis was conducted using the Kolmogorov–Smirnov test.

#### 2.3.1 Construct validity

The construct validity was evaluated using an exploratory factor analysis (EFA) to determine the optimal factor structure. The factorial structure was investigated using Generalised Least Squares factoring (66) with OBLIMIN rotation. (67) The quality of the factor analysis models was assessed with the Kaiser–Meyer–Olkin (KMO) test and the Bartlett sphericity test. The KMO measures the degree of multicollinearity and ranges from 0 to 1 (with an optimal range of >0.50–0.60) (68). We established the optimal number of factors based on Kaiser’s eigenvalue criterion (eigenvalue ≥1), evaluation of the scree plot (69), parallel analysis (70), and exploratory graph analysis (EGA); (71) and by choosing stable factors (more than 2 items per factor, lowest number of cross-loadings). These, parallel analysis and EGA, were executed using the psych and EGAnet R packages (72, 73). To evaluate the fit of the model to the data, a semi-confirmatory parallel analysis was conducted utilising various fit indices. The Root Mean Square Error of Approximation (RMSEA) was calculated, with a 90% Confidence Interval (CI). Typically, RMSEA values up to 0.08 are considered to indicate a reasonable fit to the data, with values closer to 0.05 or below suggesting a good fit. The Tucker-Lewis Index (TLI) was also determined. This index compares the fit of the proposed model to a null or baseline model. TLI values approaching or exceeding 0.95 are commonly viewed as indicative of an excellent fit to the data. The Bayesian Information Criterion (BIC) was computed as an additional measure of fit. Lower BIC values (more negative) are generally preferred, indicating a model that better captures the underlying data structure with fewer parameters. Lastly, the model’s goodness of fit was assessed using the chi-square test. A non-significant chi-square value suggests that the observed and expected covariances are not substantially different, indicating a suitable model fit.

Finally, items were selected in such a way as to preserve the theoretical structure in order to ensure the content validity of the test. For EFA, a factor loading greater than 0.4 was considered necessary for the item’s inclusion in each factor (74).

An additional measure to assess the appropriateness of the proposed factorial solution involved the use of alternative models (1, 2, 3 factors). For this purpose, fit measures, and the percentage of explained variance were analysed. Furthermore, the distribution of factors and their respective factorial weights will be presented.

#### 2.3.2 Floor and ceiling effect

The floor and ceiling effect were evaluated by calculating the percentage of patients who obtained the minimum or maximum possible scores. If at least 15% of the patients achieved the minimum/maximum score, a floor/ceiling effect was considered to be present (75).

#### 2.3.3 Concurrent validity

The concurrent validity was measured using Pearson correlations between BioPMovQ and the other disability and psychological measures. A value <0.30 was considered a low correlation, 0.30–0.60 a moderate correlation and > 0.60 a strong correlation (75).

1. Disability. Disability was assessed with the Spanish version of the GCPS, which has been employed to measure the degree of interference from chronic pain in activities of daily living. The GCPS consists of 8 items with response options in 11-point Likert format, with a total range of 0–70 points. The scale has 2 sub-scales, one measuring pain intensity and the other disability, and grades disability into moderate and severe levels. The Spanish version of this scale has demonstrated good internal consistency (Cronbach α, 0.87) (40).
2. Pain intensity. Self-reported pain intensity was assessed with the NPRS (0–10/10). On this scale, a score of 0 indicates “no pain” while a score of 10 indicates “maximum possible pain intensity” (76).
3. Pain catastrophism. To measure the level of pain catastrophising, we employed the Spanish version of the PCS, which has demonstrated adequate internal consistency (Cronbach’s α, 0.79) and test–retest reliability (ICC, 0.84) (77). The scale contains 13 items subdivided into 3 domains: rumination (constant worry and inability to inhibit pain-related thoughts, 4 items), magnification (exaggeration of the unpleasantness of pain, 3 items), and hopelessness (loss of hope for achieving something or for some physical and/or psychological aspect detrimental to health to disappear, 6 items) (77).
4. Chronic pain self-efficacy. The level of self-efficacy was assessed using the Spanish version of the CPSS, which has acceptable psychometric properties for assessing perceived self-efficacy and the ability to cope with the consequences of chronic pain (Cronbach’s α, 0.91) (78). The version consists of 19 items subdivided into 3 dimensions: self-efficacy in coping with symptom control, self-efficacy in pain management and self-efficacy in physical function. The final score ranges from 0 to 190, and the total score is obtained with the sum of the 3 dimensions, with higher scores indicating higher self-efficacy (48).
5. Fear of movement. To measure fear of movement, we employed the Spanish version of the TSK-11, which has adequate psychometric properties and good internal consistency (Cronbach’s α, 0.81) (42). The scale consists of 2 subscales, one related to fear of physical activity and the other related to fear of harm. Each of the 11 items was scored from 1–4 (1 = “strongly disagree,” 2 = “disagree,” 3 = “agree,” 4 = “strongly agree”), for total scores ranging from 11 to 44, with higher scores indicating greater fear of movement (42).

#### 2.3.4 Reliability

Internal consistency was measured with Cronbach’s α and item-total correlation coefficients. The internal consistency was considered appropriate when the α coefficient was ≥0.70 (79). We also computed McDonald’s coefficient ω (total) using the psych R package (73). This is one index recommended as an alternative to Cronbach’s α (80).

We examined the test–retest reliability using the ICC and considered that values <0.50 indicated poor reliability, 0.50–0.75 indicated moderate reliability, 0.75–0.90 indicated good reliability and > 0.90 indicated excellent reliability (81).

Measurement error was expressed as a standard error of the mean (SEM), which was calculated using the formula SEM = SD *√(1 − ICC), in which SD is the standard deviation of the values from all participants (82, 83).

The minimum detectable change (MDC) was calculated to establish whether the magnitude of change observed between the 2 measures (separated by 7–8 days) reflected real change and not just measurement error. The MDC at the 95% confidence interval (MDC_95_) was calculated as SEM × 2^−^√ × 1.96 (82).

#### 2.3.5 Discriminant validity

Discriminant validity analysis of the BioPMovQ was employed to assess varying degrees of pain-related motor and functional impairment. As a criterion variable, the disability sub-scale from the GCPS was utilised, which gauges the extent of pain interference on daily activities. Scores ranging from 17 to 24 are classified as moderately limiting interference, while those from 25 to 40 indicate severely limiting interference. In this analysis, we will classify the participants into subclinical, moderate, and severe levels of pain-related motor and functional impairment.

The Kruskal-Wallis H test and the Mann–Whitney test were applied to discern differences between the levels of motor and functional impairment linked to pain. Furthermore, the area under the receiver operating characteristic curve was evaluated to determine the proportion of patients accurately classified across different levels. The highest value for this metric is 1, indicating optimal diagnostic utility. Diagnostic accuracy is deemed excellent for values ranging from 0.9 to 1, very good for 0.8 to 0.9, good for 0.7 to 0.8, fair for 0.6 to 0.7, and poor for 0.5 to 0.6. Any value below 0.5 renders the test non-informative (84). The optimal cutoff point between levels of motor and functional impairment associated with pain was determined using the Youden index (85). Additionally, for each score, diagnostic test indicators such as sensitivity, specificity, negative predictive value, and positive predictive value were calculated.

## 3 Results

### 3.1 Content validity analysis

In the expert analysis of the content, 2 items were eliminated (“a. Physical activity can be counterproductive for my problem” and “b. Did cardiovascular exercise such as walking or cycling to reduce my pain”) because the score according to Aiken’s V was <0.70, and there were numerous comments questioning the specificity and usefulness of these items.

A total of 25 items were validated, with a range of 0.70–0.95 according to Aiken’s V. All theoretical constructs were validated by the expert committee.

Table 1 shows the Aiken V values for each item, the theoretical constructs and the general comments of the expert committee.

**Table 1:** Aiken V values for each item, the theoretical constructs and the general comments of the expert committee.

Content analysis by expert judges (*n* = 11)				
Theoretical factors and items	Relevance	Comprehensiveness	Comprehensibility	Comments
TF. Movement avoidance behaviours	0.9 (0.71 to 0.97)	1 (0.83 to 1)	0.95 (0.76 to 0.99)	
The pain increases if I perform more movements during the day	0.75 (0.53 to 0.88)	0.7 (0.48 to 0.85)	0.8 (0.58 to 0.91)	Reviewer 3. This can be an avoidance belief or an experience
Certain movements worsen my problem, and I avoid making them	0.9 (0.71 to 0.97)	0.95 (0.76 to 0.99)	0.85 (0.64 to 0.95)	Reviewer 7. This item appears to be redundant considering the previous one
Physical activity can be counterproductive for my problem	0.7 (0.48 to 0.85)	0.6 (0.38 to 0.78)	0.55 (0.34 to 0.88)	Reviewer 2, 4. Redundancy Reviewer 6. The word “problem” is very general, and the patients might not relate it to the pain specifically
The less I move the area that hurts, the better I will recover	0.8 (0.58 to 0.91)	0.85 (0.64 to 0.95)	0.9 (0.71 to 0.97)	
I avoid performing certain movements that can injure me	0.85 (0.64 to 0.95)	0.95 (0.76 to 0.99)	0.9 (0.71 to 0.97)	
My work activity worsens my pain	0.8 (0.58 to 0.91)	0.85 (0.64 to 0.95)	0.85 (0.64 to 0.95)	Revisor 10. I do not consider this is an avoidance belief
TF. Self-efficacy for physical activity	1 (0.83 to 1)	1 (0.83 to 1)	1 (0.83 to 1)	
I can perform a therapeutic exercise programme to reduce the pain	0.9 (0.71 to 0.97)	0.95 (0.76 to 0.99)	0.9 (0.71 to 0.97)	
I can perform a therapeutic exercise programme, although the symptoms increase slightly	0.95 (0.76 to 0.99)	0.95 (0.76 to 0.99)	1 (0.83 to 1)	
I can perform daily life activities that are physical demanding	0.8 (0.58 to 0.91)	0.85 (0.64 to 0.95)	0.85 (0.64 to 0.95)	Reviewer 1. There are many types of daily life activities. It might be interesting to add some examples
I can perform a physical exercise programme despite fatigue symptoms appearing	0.8 (0.58 to 0.91)	0.85 (0.64 to 0.95)	0.75 (0.53 to 0.88)	Reviewer 8, 9. Redundancy
I can perform work activities that are physical demanding	0.9 (0.71 to 0.97)	0.8 (0.58 to 0.91)	0.85 (0.64 to 0.95)	
TF. Physical discomfort	0.9 (0.71 to 0.97)	0.9 (0.71 to 0.97)	0.95 (0.76 to 0.99)	Reviewer 5. There are several items in this factor that could be in the section of avoidance beliefs and behaviours
When I have pain, I avoid moving to feel better	0.8 (0.58 to 0.91)	0.85 (0.64 to 0.95)	0.85 (0.64 to 0.95)	
When I have pain, I lie down to try to make the pain go away	0.95 (0.76 to 0.99)	0.95 (0.76 to 0.99)	0.9 (0.71 to 0.97)	
Due to the pain, the movements I make are uncoordinated and lack fluidity	1 (0.83 to 1)	0.8 (0.58 to 0.91)	0.85 (0.64 to 0.95)	
I try recreational physical activities to distract myself from the pain	0.75 (0.53 to 0.88)	0.7 (0.48 to 0.85)	0.75 (0.53 to 0.88)	Reviewer 8. Only a small group of patients would be reflected in this item.
I perform body movements to reduce the pain	0.8 (0.58 to 0.91)	0.85 (0.64 to 0.95)	0.85 (0.64 to 0.95)	
I perform cardiovascular exercises such as walking and cycling to reduce my pain	0.55 (0.34 to 0.74)	0.6 (0.38 to 0.78)	0.7 (0.48 to 0.85)	Reviewer 9. This is highly technical, difficult-to-understand language. Reviewer 4. This item is not very generalizable. Reviewer 8. Only a small group of patients would be reflected in this item.
The pain stops me from maintaining a comfortable posture, and I have to constantly change position	0.8 (0.58 to 0.91)	0.7 (0.48 to 0.85)	0.75 (0.53 to 0.88)	
TF. Self-perceived functional ability	0.95 (0.76 to 0.99)	1 (0.83 to 1)	0.95 (0.76 to 0.99)	
My daily life activities tire me out	0.9 (0.71 to 0.97)	0.95 (0.76 to 0.99)	0.85 (0.64 to 0.95)	
My muscles are tense (rigid) and lack flexibility	0.7 (0.48 to 0.85)	0.85 (0.64 to 0.95)	0.85 (0.64 to 0.95)	Reviewer 3. This item does not correspond to the functional capacity concept
The movements I make are uncoordinated and jerky	0.9 (0.71 to 0.97)	0.95 (0.76 to 0.99)	0.9 (0.71 to 0.97)	
I have little strength or muscle resistance	1 (0.83 to 1)	1 (0.83 to 1)	1 (0.83 to 1)	
I have difficulty performing movements that require a lot of precision such as grasping, manipulating, or cutting objects.	0.9 (0.71 to 0.97)	0.95 (0.76 to 0.99)	0.9 (0.71 to 0.97)	
TF. Disability	1 (0.83 to 1)	1 (0.83 to 1)	1 (0.83 to 1)	Reviewer 11. It appears to me that this factor evaluates the interference of pain in activities more than the disability construct
The pain stops me from adequately performing my work activity	0.8 (0.58 to 0.91)	0.85 (0.64 to 0.95)	0.85 (0.64 to 0.95)	
The pain stops me from adequately performing my household chores	0.75 (0.53 to 0.88)	0.7 (0.48 to 0.85)	0.85 (0.64 to 0.95)	
The pain has decreased or halted my recreational and societal activities	0.95 (0.76 to 0.99)	0.9 (0.71 to 0.97)	0.85 (0.64 to 0.95)	
The pain stops me from performing physical and sport activities	0.8 (0.58 to 0.91)	0.7 (0.48 to 0.85)	0.85 (0.64 to 0.95)	

TF, Theoretical factors.

### 3.2 Characteristics of the sample

The total sample consisted of 200 participants with chronic musculoskeletal pain, 68.6% of whom were women. Table 2 presents the patients’ sociodemographic characteristics and scores on the various self-reported scales. The BioPMovQ data did not follow a normal distribution, but the instrument’s response rate was 100%. In Table 3, the descriptive statistics for each item of the BioPMovQ are presented, along with the ‘if item dropped’ metrics such as Item, Mean, SD, Skewness, Kurtosis, frequencies for 0 to 4, Item-rest correlation, Cronbach’s α, and McDonald’s ω.

**Table 2:** Sociodemographic and clinical data and scores obtained on the self-reported scale.

Sociodemographic and clinical data	Mean ± SD	Range (Min-Max)
Age (years)	48.87 ± 14.75	19–82
BDI (kg/m^2^)	25.66 ± 4.61	17.36–39.45
BioPMovQ	38.36 ± 11.07	14–64
Pain duration (months)	35.12 ± 34.02	6–156
Self-efficacy for physical activity	7.43 ± 3.51	1–16
Disability	10.04 ± 4.07	1–16
Movement fear-avoidance beliefs and behaviours	9.15 ± 2.85	1–12
Self-perceived functional ability	11.62 ± 4.94	1–20
TSK-11	27.51 ± 7.64	12–44
TSK harm	12.05 ± 4.87	3–28
TSK activity avoidance	15.53 ± 5.25	4–28
PCS	15.37 ± 10.43	1–45
PCS rumination	5.54 ± 3.97	0–16
PCS magnification	3.37 ± 2.53	0–12
PCS helplessness	6.47 ± 5.02	1–20
CPSS	137.83 ± 33.1	28–190
PSE	57.22 ± 14.34	9–80
SSE	30.81 ± 12.36	0–50
FSE	49.74 ± 11.91	8–60
GCPS	36.98 ± 13.95	6–63
GCPS pain intensity	19.41 ± 4.66	4–30
GCPS disability	17.70 ± 10.92	0–40
Numerical pain rating scale	6.52 ± 1.66	3–10
Categorical variables	n (%)	
Gender		
Women	153 (68.6)	
Men	47 (21.1)	
Employment status		
Employed	120 (53.8)	
Unemployed	18 (8.1)	
Medical leave due to disability	27 (12.1)	
Retired	35 (15.7)	
Level of Education		
Uneducated	3 (1.3)	
Primary education	26 (11.7)	
Secondary education	82 (36.8)	
University education	89 (39.9)	

BDI, Body mass index; BioPMovQ, Biobehavioural Pain and Movement Questionnaire; TSK-11, Tampa Scale of Kinesiophobia; PCS, Pain Catastrophising Scale; CPSS, Chronic Pain Self-Efficacy Scale; GCPS, Graded Chronic Pain Scale; PSE, self-efficacy in pain management; SSE, self-efficacy in coping with symptom control; FSE, self-efficacy in physical function; SD, standard deviation.

**Table 3:** Descriptive statistics.

											if item dropped	
Item	Mean	SD	Skewness	Kurtosis	Freq. 0	Freq. 1	Freq. 2	Freq. 3	Freq. 4	Item-rest correlation	Cronbach’s α	McDonald’s ω
1	2.97	1.27	−1.17	0.24	0.09	0.08	0.08	0.31	0.46	0.43	0.83	0.84
2	1.82	1.18	0.55	−0.57	0.10	0.38	0.30	0.09	0.15	0.31	0.84	0.84
3	2.45	1.34	−0.41	−1.08	0.10	0.19	0.15	0.29	0.28	0.45	0.83	0.84
4	1.80	1.30	0.38	−1.09	0.15	0.39	0.14	0.19	0.15	0.31	0.84	0.84
5	2.45	1.38	−0.40	−1.23	0.10	0.23	0.09	0.30	0.29	0.50	0.83	0.83
6	3.20	1.16	−1.55	1.49	0.06	0.06	0.07	0.27	0.56	0.49	0.83	0.84
7	1.83	1.12	0.44	−0.40	0.10	0.33	0.36	0.11	0.12	0.20	0.84	0.85
8	2.12	1.45	−0.03	−1.43	0.17	0.26	0.13	0.20	0.25	0.29	0.84	0.84
9	2.88	1.34	−1.10	−0.04	0.12	0.07	0.08	0.31	0.43	0.33	0.84	0.84
10	2.41	1.36	−0.44	−1.12	0.12	0.19	0.11	0.33	0.26	0.55	0.83	0.83
11	1.71	1.39	0.36	−1.26	0.22	0.35	0.08	0.21	0.15	0.36	0.84	0.84
12	1.95	1.09	0.31	−0.61	0.07	0.32	0.34	0.18	0.11	0.22	0.84	0.84
13	3.26	1.14	−1.73	2.13	0.06	0.06	0.03	0.29	0.57	0.31	0.84	0.84
14	3.06	1.28	−1.38	0.74	0.10	0.06	0.05	0.30	0.51	0.46	0.83	0.84
15	2.81	1.35	−0.78	−0.80	0.07	0.18	0.06	0.26	0.44	0.46	0.83	0.84
16	1.55	1.38	0.57	−0.97	0.27	0.34	0.12	0.13	0.15	0.25	0.84	0.85
17	1.81	1.09	0.37	−0.47	0.10	0.34	0.33	0.15	0.09	0.31	0.84	0.84
18	1.70	1.44	0.33	−1.21	0.28	0.22	0.20	0.13	0.18	0.35	0.84	0.84
19	2.09	1.46	−0.16	−1.45	0.20	0.22	0.07	0.31	0.20	0.57	0.83	0.83
20	2.99	1.31	−1.22	0.21	0.09	0.10	0.02	0.32	0.48	0.50	0.83	0.84
21	1.89	1.28	0.29	−0.92	0.14	0.29	0.28	0.13	0.17	0.26	0.84	0.84
22	1.98	1.11	0.49	−0.43	0.06	0.30	0.41	0.08	0.16	0.44	0.84	0.84
23	2.55	1.46	−0.60	−1.10	0.15	0.15	0.09	0.27	0.36	0.57	0.83	0.83
24	2.39	1.54	−0.42	−1.38	0.19	0.15	0.09	0.23	0.35	0.31	0.84	0.84
25	2.21	1.50	−0.23	−1.44	0.20	0.19	0.11	0.25	0.27	0.48	0.83	0.84

SD, standard deviation.

### 3.3 Exploratory factor analysis

Item statistics are presented in the Table 3. It can be seen how all items contribute to internal consistency and that skewness and kurtosis generally remain below 2. The KMO test showed an acceptable data suite for factor analysis (KMO score of 0.829), there were no multicollinearity problems, and Bartlett’s test of sphericity rejected the identity matrix null hypothesis (χ^2^ (120) = 869.88, *p* < *0*.001).

Based on these results, continuing with the EFA would be justified. Lastly, we used the generalised least squares method of factor extraction with oblimin rotation. The semi-confirmatory parallel analysis recommended between 3 and 5 factors, although the EGA analysis with 500 bootstrap iterations showed that the most repeated solutions were those with 3 to 5 factors (with proportions of 0.324, 0.376, 0.184, respectively). For theoretical reasons and in order to cover all relevant areas described in developing items section finally present a four-factor solution. The four-factor model of the BioPMovQ exhibited a favourable fit as indicated by the model fit metrics: χ^2^ (62) = 74.6, *p* = 0.131; BIC = −254; TLI = 0.967; RMSEA = 0.031 with a 95% confidence interval of 0.001–0.055.

The four-factor solution which together represented 55.79% of the total variance. The first factor (28.85% of the total variance) consisted of 4 items. The theoretical content of this factor was labelled “disability.**”** The second factor (13.55% of the total variance) consisted of 4 items and referred to self-efficacy for physical activity. The third factor (“movement fear-avoidance beliefs and behaviours”) included 3 items and accounted for 6.84% of the total variance. The fourth factor (“self-perception of functional ability”) presented 4 items and accounted for 6.82% of the total variance. The factor loadings for each item are shown in Table 4. A total of 7 items were eliminated because the factorial weight was <0.40.

**Table 4:** Observational factor analysis.

	Disability	Self-efficacy for physical activity	Movement avoidance behaviours	Self-perceived functional ability
5. The pain stops me from adequately performing my work activity	**0.84**			0.40
10. The pain stops me from adequately performing my household chores	**0.68**		0.44	0.39
24. The pain has decreased or halted my recreational and societal activities	**0.49**			
15. The pain stops me from performing physical and sport activities	**0.44**		0.35	0.41
2. I can perform a therapeutic exercise programme to reduce the pain		**0.73**		
22. I can perform work activities that are physical demanding		**0.67**		
17. I can perform a physical exercise programme despite fatigue symptoms appearing		**0.66**		
12. I can perform daily life activities that are physical demanding		**0.59**		
6. Certain movements worsen my problem, and I avoid making them	0.37		**0.90**	0.42
20. I avoid performing certain movements that can injure me	0.40		**0.47**	0.45
1. The pain increases if I perform more movements during the day			**0.45**	0.39
23. I have little strength or muscle resistance	0.60			**0.72**
25. I have difficulty performing movements that require a lot of precision such as grasping, manipulating, or cutting objects.	0.34			**0.70**
19. I have difficulty performing daily life activities such as walking fast or climbing stairs	0.41			**0.67**
14. My muscles are tense (rigid) and lack flexibility			0.37	**0.51**
11. My daily life activities tire me out				**0.45**

Method-GLS_Oblimin. Bold values indicate factor analysis results >0.40.

The alternative models analysed do not offer a suitable factorial solution, as their fit metrics are not acceptable. Additionally, several items exhibit factorial weights below 0.40, and the percentage of variance explained by each of these alternative models is significantly lower than that of the proposed factorial solution (Tables 5, 6).

**Table 5:** Comparations fit measures for factor models.

		RMSEA 90% CI				Model test			
Model	RMSEA	Lower	Upper	TLI	BIC	χ^2^	df	*p*	% of variance
4 Factors	0.031	0.001	0.055	0.967	−254	74.6	62	0.131	55.79%
3 Factors	0.056	0.038	0.074	0.895	−274	124	75	< 0.001	48.9%
2 Factors	0.066	0.051	0.082	0.857	−303	168	89	< 0.001	42.12%
1 Factors	0.106	0.093	0.119	0.639	−213	338	104	< 0.001	28.57

RMSEA, Root Mean Square Error of Approximation; CI, Confidence Interval; TLI, Tucker-Lewis Index; BIC, Bayesian Information Criterion; χ^2^, Chi-squared Test; df, Degrees of Freedom; % of Variance, Percentage of Variance Explained; 90% CI, 90% confidence interval.

**Table 6:** Factorial structure using alternative models to the proposed factorial structure.

	1 Factor Model	2 Factors Model		3 Factors Model		
	1	1	2	1	2	3
5. The pain stops me from adequately performing my work activity	0.63	0.68		0.71		
10. The pain stops me from adequately performing my household chores	0.65	0.66		0.63		
24. The pain has decreased or halted my recreational and societal activities	0.37**	0.42		0.46		
15. The pain stops me from performing physical and sport activities	0.51	0.52		0.49		
2. I can perform a therapeutic exercise programme to reduce the pain	0.33**	0.21**			0.71	
22. I can perform work activities that are physical demanding	0.38**	0.27**			0.67	
17. I can perform a physical exercise programme despite fatigue symptoms appearing	0.26**	0.14**			0.67	
12. I can perform daily life activities that are physical demanding	0.18**		0.58		0.58	
6. Certain movements worsen my problem, and I avoid making them	0.57	0.58				0.922
20. I avoid performing certain movements that can injure me	0.55	0.55				0.49
1. The pain increases if I perform more movements during the day	0.47	0.45				0.45
23. I have little strength or muscle resistance	0.71	0.72				0.33**
25. I have difficulty performing movements that require a lot of precision such as grasping, manipulating, or cutting objects.	0.55	0.55		0.56		
19. I have difficulty performing daily life activities such as walking fast or climbing stairs	0.62	0.60		0.61		
14. My muscles are tense (rigid) and lack flexibility	0.48	0.47		0.43		–
11. My daily life activities tire me out	0.41	0.40		0.40		

**Items recommended for removal due to factor loadings below 0.40.

There was no floor or ceiling effect. Two patients scored 18 points, which is the minimum possible (0.01%), and only 1 patient scored the maximum (0.001%/72 points).

### 3.4 Concurrent validity

Overall, the BioPMovQ total score presented moderate magnitude correlations with most of the psychological and disability variables. With respect to the BioPMovQ subscales, the correlations were small to moderate in magnitude. Table 7 shows the correlations between the BioPMovQ and its subscales with all the assessed self-reported scales.

**Table 7:** Concurrent validity of the BioPMovQ.

Concurrent validity	BioPMovQ				
	Total score	Disability	Self-efficacy for physical activity	Movement avoidance behaviours	Self-perceived functional ability
GCPS	**0.52	**0.55	**0.27	**0.39	**0.38
GCPS disability	**0.51	**0.54	**0.28	**0.34	**0.35
GCPS pain intensity	**0.44	**0.40	*0.17	**0.38	**0.33
NPRS	**0.31	*0.17	*0.15	**0.28	**0.26
TSK-11	**0.60	**0.45	**0.22	**0.53	**0.54
TSK harm	**0.51	**0.25	**0.51	**0.32	**0.38
TSK activity avoidance	**0.32	**0.35	*0.14	**0.34	**0.37
PCS	**0.44	**0.43	*0.18	**0.32	**0.38
PCS rumination	**0.39	**0.37	**0.22	**0.28	**0.33
PCS magnification	**0.32	**0.31	**0.16	**0.24	**0.26
PCS helplessness	**0.42	**0.44	0.13	**0.31	**0.39
CPSS	**−0.31	**−0.32	**−0.44	**−0.22	**−0.21
PSE	**−0.26	**−0.27	**−0.37	**−0.19	**−0.19
SSE	**−0.26	**−0.29	**−0.40	**−0.21	**−0.18
FSE	**−0.27	**−0.25	**−0.34	*−0.16	**−0.18

BioPMovQ, Biobehavioural Pain and Movement Questionnaire; NPRS, Numerical Pain Rating Scale; TSK, Tampa Scale of Kinesiophobia; PCS, Pain Catastrophising Scale; CPSS, Chronic Pain Self-Efficacy Scale; PSE, self-efficacy in pain management; SSE, self-efficacy in coping with symptom control; FSE, self-efficacy in physical function; GCPS, Graded Chronic Pain Scale.

### 3.5 Reliability

The internal consistency of the BioPMovQ was Cronbach’s Alpha = 0.82 and McDonald’s *ω* = 0.83, with its 4 subscales showing an internal consistency of 0.64–0.75. To assess the instrument’s test–retest reliability, 51 patients (74.5% women; age, 47.74 ± 14.71 years) re-took the scale 10.13 ± 12.52 days later. According to the ICC, the scale’s stability over time was excellent, with an MDC_95_ of 9.50. Table 8 shows the descriptive statistics and results of the test–retest reliability and responsiveness analysis for the BioPMovQ and its subscales. The highest correlations with the other instruments of the global scale were presented with the GCPS and the TSK-11 and were lower with the NPRS.

**Table 8:** Reliability analysis.

	McDonald’s ω	Cronbach’s alpha	Mean ± SD		ICC (95% CI)	SEM	MDC_90_	MDC_95_
			Test 1	Test 2				
BioPMovQ	0.83	0.82	36.06 ± 10.7	38.16 ± 8.03	0.86 (0.76 to 0.91)	3.43	8.04	9.50
Disability	0.71	0.70	9.54 ± 4	10.26 ± 3.55	0.86 (0.77 to 0.91)	1.41	3.29	3.91
Self-efficacy for physical activity	0.75	0.75	6.16 ± 2.67	6.88 ± 2.33	0.88 (0.80 to 0.93)	0.87	2.03	2.41
Movement fear-avoidance beliefs	0.65	0.64	8.54 ± 3.27	8.69 ± 2.95	0.86 (0.77 to 0.91)	1.16	2.71	3.22
Self-perceived functional ability	0.74	0.73	11.56 ± 4.33	12.26 ± 3.23	0.83 (0.72 to 0.90)	1.58	3.68	4.37

BioPMovQ, Biobehavioural Pain and Movement Questionnaire; SD, standard deviation; ICC, intraclass correlation coefficient; MDC_90_, minimal detectable change at the 90% confidence level; MDC_95_, minimal detectable change at the 95% confidence level; SEM, standard error of the mean.

### 3.6 Discriminant validity

**Figure 1:**
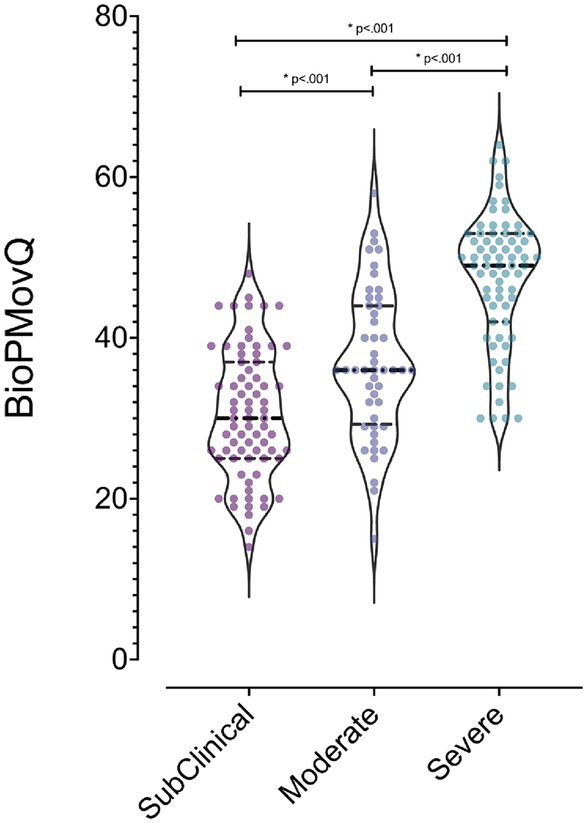
Differences in functional and motor alterations levels related to pain as measured by the BioPMovQ. *Indicates statistical significance (*p* < .001) and the 95% confidence interval considered for comparisons between variables using the Mann–Whitney U test.

Utilizing the Kruskal-Wallis test, significant differences in BioPMovQ were observed among levels of pain-related motor and functional impairment (*H* = 64.54, *p* < 0.001). Subsequent Mann–Whitney tests indicated disparities between: (a) individuals subclinical and those with moderate pain-related motor and functional impairment (*U* = 1340.50, *p* = 0.003), (b) those subclinical and those with severe pain-related motor and functional impairment (*U* = 706, *p* < 0.001), and (c) moderate versus severe levels (*U* = 803.50, *p* < 0.001). The rank averages consistently reflected a pattern where the scores increased with the severity of pain-related motor and functional impairment, suggesting a clear distinction in BioPMovQ scores based on the level. In Figure 1, a violin plot representation of the various levels of the BioPMovQ based on pain-related motor and functional impairment can be observed.

**Figure 2:**
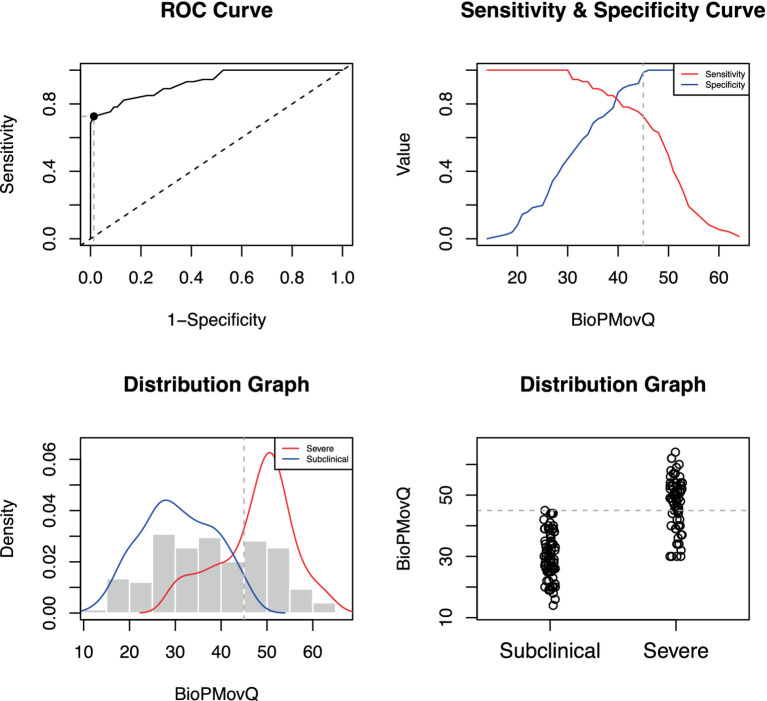
Optimal cut-off point between levels of BioPMovQ (Subclinical vs. Severe). A ROC (receiver operating characteristic) curve that represents the sensitivity of a diagnostic test that produces continuous results, depending on false positives (complementary to specificity), for different cut-off points, the image where the cut-off point at which the highest sensitivity and specificity is achieved and finally, a subclinical and moderate sample distribution graph.

**Figure 3:**
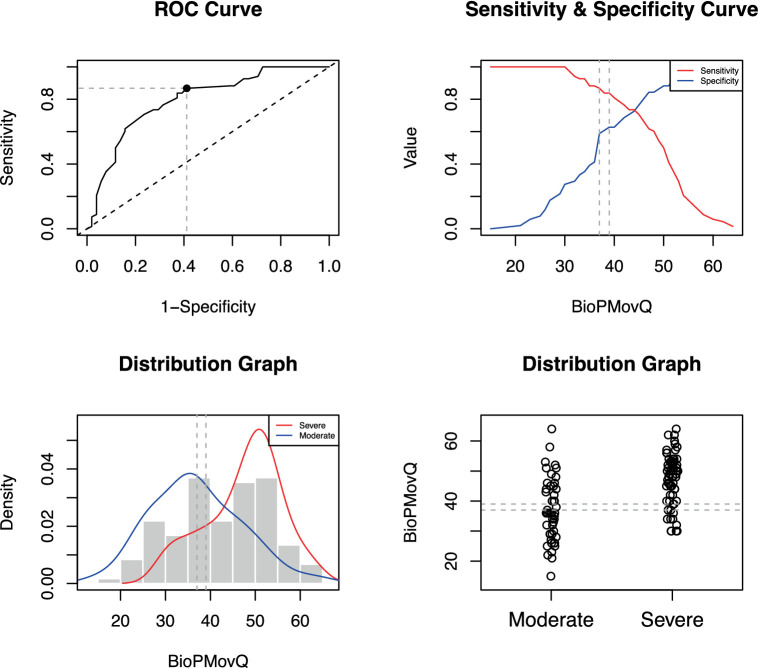
Optimal cut-off point between levels of BioPMovQ (Moderate vs. Severe). A ROC (receiver operating characteristic) curve that represents the sensitivity of a diagnostic test that produces continuous results, depending on false positives (complementary to specificity), for different cut-off points, the image where the cut-off point at which the highest sensitivity and specificity is achieved and finally, a subclinical and moderate sample distribution graph.

The BioPMovQ demonstrated robust diagnostic precision in discerning patients at the severe level, evidenced by high specificity and sensitivity. In terms of the moderate level, sensitivity was satisfactory, while specificity was commendable. Table 9 delineates the diagnostic precision, including the salient cut-off points. The optimal cutoffs were determined to be <37 for subclinical, ≥37 for moderate, and ≥ 45 for severe levels. For the moderate level, sensitivity was 0.87 with a 95% CI of [0.76–0.84], while specificity stood at 0.59 with a 95% CI of [0.44–0.62]. For the severe level, sensitivity and specificity were 0.72 ([0.61–0.82]) and 0.98 ([0.92–1]), respectively. Figures 2, 3 further elucidate these findings. To determine the optimal cut-off points and conduct sensitivity and specificity analyses, the R package named OptimalCutpoints was used (86).

**Table 9:** Diagnostic accuracy results and all optimal cut-off points of BioPMovQ.

	Subclinical	Moderate	Severe
Mean ± SD	30.73 ± 8.03	36.85 ± 9.27	47.49 ± 8.15
95% CI	[28.91–32.54]	[34.16–39.86]	[45.56–49.42]
Median (25th percentile; 75th percentile)	30 (P25 = 25; P75 = 38)	36 (P25 = 29.25; P75 = 44)	49 (P25 = 42; P75 = 53)
Cases, N (%)	78 (39.59%)	48 (24.37%)	71 (36.04%)
Optimal cuff-off point	<37	≥37	≥45
Sensitivity (95% CI)	–	0.87 [0.76–0.84]	0.72 [0.61–0.82]
Specificity (95% CI)	–	0.59 [0.44–0.62]	0.98 [0.92–1]
Positive predictive value (95% CI)	–	0.73 [0.61–0.75]	0.98 [0.90–0.98]
Negative predictive value (95% CI)	–	0.78 [0.62–0.74]	0.78 [0.68–0.99]

SD, standard deviation; 95% CI, confidence interval.

## 4 Discussion

The BioPMovQ is an instrument designed to assess (from a biobehavioural perspective) pain related to functional and motor impairments in patients with musculoskeletal pain. This study provides evidence on the psychometric properties of the BioPMovQ, including its content validity, factor analysis, internal consistency, test–retest reliability and concurrent validity, which suggest that this instrument meets the recommended minimum standards for patient-centered measures proposed by the International Society for Quality of Life Research (87). Overall, the findings of this research show that the BioPMovQ has adequate psychometric properties, showing good validity and reliability for the assessment of the designed construct.

The results of the study indicate that the BioPMovQ has good content validity as assessed by experts, indicating that the questions in the questionnaire have adequate relevance, completeness and comprehensibility and are suitable for measuring the construct of pain related to functional and motor impairments from a biobehavioural perspective in the population with musculoskeletal pain. The BioPMovQ also met the criteria proposed by Patrick et al. (88) for determining good content validity: a correct qualitative phase of instrument development and construction and evidence that identifies adequate understanding of the instrument (88). In the content analysis phase, the experts validated 5 theoretical sub-constructs of the initial instrument; in the exploratory factor analysis, they obtained a 4-factor solution that together accounted for 55.79% of the total variance. The values obtained in the KMO index indicate that the instrument has a good level of multicollinearity between items (89). Each of the subscales had 3–5 items, as recommended by a number of authors (89). The factor loadings of 17 of the 18 items were ≥ 0.44, which are considered strong (74), increasing the solidity of the obtained factor structure. Another positive aspect is that a 5-point Likert scale was employed, which is a good option when the data follow a normal distribution, as in our case (67).

Lastly, the following 4 factors or subscales were included: disability; self-efficacy for physical activity; movement avoidance behaviours; self-perceived functional ability. For 2 of the factors, however, the initial theoretical name had to be redefined, possibly because 7 items were eliminated during the exploratory factor analysis, and 3 items were distributed differently from the initial theoretical assumption.

The BioPMovQ has been designed and constructed from a biobehavioural perspective, which implies that the biological, psychological (affective, cognitive) and social aspects are interrelated (90), thereby providing a broader view in understanding the impact of pain on movement and function in patients with chronic musculoskeletal pain. It makes sense to employ global scoring for decision making using the biobehavioural paradigm.

With its various subscales, the BioPMovQ provides an innovative and comprehensive clinical assessment, with 4 measured factors that can be considered determinants for the functional approach to patients with chronic pain, for which other instruments are not currently integrated into a single construct. The physical activity self-efficacy subscale assesses the patient’s confidence in their ability to perform physical activities despite pain. The disability subscale assesses the impact of pain on the performance of work and daily living activities. The movement avoidance behaviour subscale assesses the individual’s tendency to avoid certain activities due to pain. The self-perceived functional ability subscale assesses the individual’s ability to perform specific tasks related to physical exertion.

Findings related to concurrent validity show that the BioPMovQ presents low/moderate correlations with instruments measuring chronic pain self-efficacy, pain catastrophising, pain intensity, disability and kinesiophobia, the latter 2 correlations being the highest recorded (*r* = 0.52 and *r* = 0.60, respectively). These results describe possible relationships with other constructs but also demonstrate that the BioPMovQ and its subscales measure different aspects and rule out the possibility that the instrument is repetitive or redundant with respect to other instruments. The internal consistency of the BioPMovQ was good (Cronbach’s *α* = 0.82; McDonald’s *ω* = 0.83); that of most of the subscales was also acceptable (Cronbach’s *α*, >0.70), except for the subscale of avoidance motor behaviours (Cronbach’s *α*, 0.64), which were poor. This subscale includes only 3 items was the shortest subscale and that could have affected internal consistency.

The test–retest reliability of the BioPMovQ was good (ICC of 0.86), as were those of all the subscales (ICC > 0.83). A mean of separation of 10.13 ± 12.52 days between the 2 measurements was employed to prevent patients from recalling their previous responses and avoid strong fluctuations in their clinical status. This period was adequate, considering that a period of 2–14 days between measurements is considered acceptable for assessing test–retest reliability (91). Considering the BioPMovQ’s total score, the SEM and the MDC_90_ presented relatively low values (3.43 and 8.04, respectively), a relevant aspect given that the purpose of the MDC is to detect real changes that are outside the measurement error (92), with smaller results indirectly indicating that the measure is stable in repeated measurements over time.

As for the floor/ceiling effect, only 2 patients (0.01%) obtained the minimum score, while 1 patient (0.001%) obtained the maximum score, which indicates an absence of the floor/ceiling effect (75). The 100% BioPMovQ response rate could have been due to several reasons, including the rigorous process employed for developing the items and the adequate understanding demonstrated in the pilot test with patients. Another factor to consider is the instrument’s brevity. Other authors have reported an association between the questionnaire’s high response rate and length, given that longer questionnaires have lower response rates (93).

### 4.1 Clinical implications

The introduction of the BioPMovQ represents a significant advancement in the assessment and management of chronic musculoskeletal pain from a biobehavioural perspective. This psychometrically validated tool offers a comprehensive approach to understanding how pain impacts patient functionality and motor behaviour, acknowledging the interplay of biological, psychological, and social factors.

To our knowledge, this is the first instrument developed and psychometrically validated to assess the construct of pain related to functional and motor impairment from a biobehavioural perspective, integrating 4 subscales, 3 of which (self-efficacy for physical activity, disability, movement avoidance behaviours) already have instruments assessing similar constructs. Self-perceived functional ability have not however been sufficiently reported in the literature on chronic musculoskeletal pain, highlighting one the major advantages of the BioPMovQ: the integration of several sub-constructs into a short questionnaire, an important advantage for its clinical use in patients with chronic musculoskeletal pain, given its a broad view of pain-related functional and motor impairments. In certain contexts, the use of multiple questionnaires can be a limitation; as Vickers suggests, this can lead to excessive patient drop-out, undue burden of data management and difficulties with interpreting the results (94). In the context of musculoskeletal pain research and clinical implications, the BioPMovQ could be a solution to the overuse of self-registration.

One of the key contributions of the BioPMovQ to clinical practice is its ability to facilitate a more personalised approach to treating musculoskeletal pain. By assessing various dimensions related to pain and its influence on motor behaviour, clinicians can identify specific therapeutic targets for each patient, such as improving self-efficacy for physical activity, reducing movement avoidance behaviours, and enhancing perceived functional capacity. This customization of care aims not only to optimise therapeutic outcomes but also to improve the quality of life for patients.

Furthermore, the BioPMovQ holds potential as a valuable tool in future research on musculoskeletal pain. Its application in longitudinal studies could provide new insights into how specific therapeutic interventions, such as exercise programs or psychosocial coping strategies, affect the experience of pain and its functional consequences over time. Additionally, it could facilitate the exploration of the dynamics between biological and behavioural components of pain, paving the way for more effective biobehavioural interventions.

Another relevant clinical implication of the BioPMovQ lies in its capacity to serve as a means of communication among different healthcare professionals involved in the management of musculoskeletal pain. By providing a common language to describe the complexity of pain and its effects on mobility and functionality, it enables more effective interdisciplinary collaboration, essential for comprehensive therapeutic approaches.

### 4.2 Limitations and future studies

Although the BioPMovQ has demonstrated promising psychometric properties, we acknowledge several limitations that underline the need for future research. First, the current study focused on a population with chronic musculoskeletal pain. It is essential to replicate these findings in populations with acute pain conditions and across cultural contexts to validate the instrument’s universality.

Secondly, while exploratory factor analysis provided a solid factorial structure for the BioPMovQ, confirmation of this structure through confirmatory factor analysis in independent samples is crucial. This step will not only reinforce the construct validity of the instrument but also its applicability across different populations and contexts.

Furthermore, item response analysis (IRT) offers an opportunity to examine the utility of each item across the construct measurement spectrum. This approach could provide valuable insights into the BioPMovQ’s sensitivity to clinically meaningful changes, helping to further refine the instrument to capture crucial aspects of pain and movement from a biobehavioural perspective.

An exhaustive evaluation of the DIF and the measurement invariance of the instrument through variables such as sex remains to be done. On a tentative basis, we ran the Mantel–Haenszel procedure with purification and found that items 14, 17 and 25 appeared to be marked with DIF. It is important to verify this result in a larger sample and better balanced by sex.

A particular area of interest is the exploration of individual and group differences in responses to the BioPMovQ. Investigating measurement invariance across demographic groups, such as gender, age, and pain type, will determine if the instrument consistently interprets across diverse groups, ensuring its fairness and accuracy in measurement across different populations.

Finally, longitudinal studies employing the BioPMovQ to assess the efficacy of specific interventions, such as therapeutic exercise programs or psychosocial interventions, could provide additional evidence on the instrument’s sensitivity to changes over time. This is essential for confirming the BioPMovQ’s utility in monitoring treatment progress and assessing outcomes in patients with musculoskeletal pain.

## 5 Conclusion

This study provides evidence for the psychometric properties of the BioPMovQ. The fact that it showed good content validity, internal consistency and test–retest reliability suggests that it is a reliable and accurate instrument for assessing the relationship between pain and functional impairements and movement. In addition, the identification of 4 subscales provides a more detailed and accurate assessment tool for health professionals involved in the care of patients with chronic musculoskeletal pain. We consider the BioPMovQ to be an instrument that can be used in future clinical research.

## Data availability statement

The raw data supporting the conclusions of this article will be made available by the authors, without undue reservation.

## Ethics statement

The studies involving humans were approved by Centro Superior de Estudios Universitarios La Salle. The studies were conducted in accordance with the local legislation and institutional requirements. The participants provided their written informed consent to participate in this study.

## Author contributions

RT: Conceptualization, Formal analysis, Investigation, Methodology, Writing – original draft, Writing – review & editing. AP-A: Writing – review & editing. JP-M: Formal analysis, Investigation, Methodology, Writing – review & editing. DM-M: Investigation, Writing – review & editing. FM-R: Writing – review & editing. IR-D: Writing – review & editing. MS: Formal analysis, Writing – review & editing. MG-A: Investigation, Writing – original draft, Writing – review & editing.
